# Extracellular Vesicles Arising from Apoptotic Cells in Tumors: Roles in Cancer Pathogenesis and Potential Clinical Applications

**DOI:** 10.3389/fimmu.2017.01174

**Published:** 2017-09-22

**Authors:** Catherine Lynch, Maria Panagopoulou, Christopher D. Gregory

**Affiliations:** ^1^MRC Centre for Inflammation Research, Queen’s Medical Research Institute, University of Edinburgh, Edinburgh, United Kingdom

**Keywords:** extracellular vesicle, apoptosis, exosome, ectosome, cancer pathogenesis, wound healing, regeneration

## Abstract

It is known that apoptotic cells can have diverse effects on the tumor microenvironment. Emerging evidence indicates that, despite its renowned role in tumor suppression, apoptosis may also promote oncogenic evolution or posttherapeutic relapse through multiple mechanisms. These include immunomodulatory, anti-inflammatory, and trophic environmental responses to apoptosis, which drive tumor progression. Our group has introduced the term “onco-regenerative niche (ORN)” to describe a conceptual network of conserved cell death-driven tissue repair and regeneration mechanisms that are hijacked in cancer. We propose that, among the key elements of the ORN are extracellular vesicles (EVs), notably those derived from apoptotic tumor cells. EVs are membrane-delimited subcellular particles, which contain multiple classes of bioactive molecules including markers of the cell from which they are derived. EVs are implicated in an increasing number of physiological and pathological contexts as mediators of local and systemic intercellular communication and detection of specific EVs may be useful in monitoring disease progression. Here, we discuss the mechanisms by which EVs produced by apoptotic tumor cells—both constitutively and as a consequence of therapy—may mediate host responsiveness to cell death in cancer. We also consider how the monitoring of such EVs and their cargoes may in the future help to improve cancer diagnosis, staging, and therapeutic efficacy.

## Introduction: Apoptosis and Oncogenesis

Apoptosis plays important roles in regulating cell populations during ontogeny and in adult tissues. Emerging evidence indicates that, not only is apoptosis responsible for the well-established deletion of cells during organ sculpting (for example, removal of the cells of the interdigital webs during limb development of mammals) but also for stimulating the proliferation of cells in neighboring compartments, a process termed compensatory proliferation or apoptosis-induced proliferation (1–3). In adults, apoptosis is prominent in controlling responses to infection and other inflammatory stimuli and in cyclical turnover of tissues ranging from the lactating/post-lactating mammary gland to the turnover of epithelia. In the steady state in which cell gain is balanced by cell loss, apoptosis provides important tissue homeostatic signals, ensuring the safe, non-phlogistic removal of billions of cells daily.

In malignant disease, the homeostatic cell birth/cell death balance becomes dysregulated through acquisition of oncogenic mutations that lead to net expansion of transformed cell populations. In addition to the loss of normal constraints on proliferation, the capacity to evade apoptosis and survive inappropriately facilitates oncogenesis. Thus, mutations affecting genes that promote cell survival such as antiapoptotic Bcl-2 family members and molecules active in the PI3K/Akt pathway complement those that promote cell proliferation (4). In the face of frequent mitoses, tumors that grow slowly—a typical example being basal cell carcinoma—display relatively high rates of apoptosis [high apoptotic index (AI)] (5). Less intuitively, aggressive, rapidly growing tumors also display high AIs. Indeed, close association between high AI and high proliferative rate has been reported for multiple aggressive cancers, including colorectal carcinoma, bladder, lung and breast cancers, leukemia, and lymphoma (6). To place these observations in perspective, it has long been known that in Burkitt’s lymphoma, for example, which displays evidence of both high mitotic and apoptotic indices in standard histological sections, a substantial proportion (around 70%) of the proliferating cells die (7). This accords with the principle that apoptosis is often prominent in association with proliferation in normal tissues.

Constitutive apoptosis in growing tumors is likely to be caused by multiple stresses characteristic of rapidly expanding tissues, cell growth outpacing (i) supply of nutrients and oxygen and (ii) removal of potentially toxic metabolites. Additional causes of constitutive apoptosis include survival pathway “insufficiency” (caused by genetic mutation), antitumor immunity, and cell competition effects. Apoptosis of tumor cells is also the basis for the effectiveness of anticancer chemotherapies and radiotherapy. Recent evidence indicates that microenvironmental tissue repair and regenerative responses to tumor cell apoptosis are critically important in promoting net tumor growth and posttherapeutic tumor repopulation/relapse (8–11). We propose that extracellular vesicles (EVs) play key roles in potentiating the microenvironmental effects of apoptosis in tumors.

## EVs: Classes, Cargoes, and Functional Properties in Cancer

**Figure 1:**
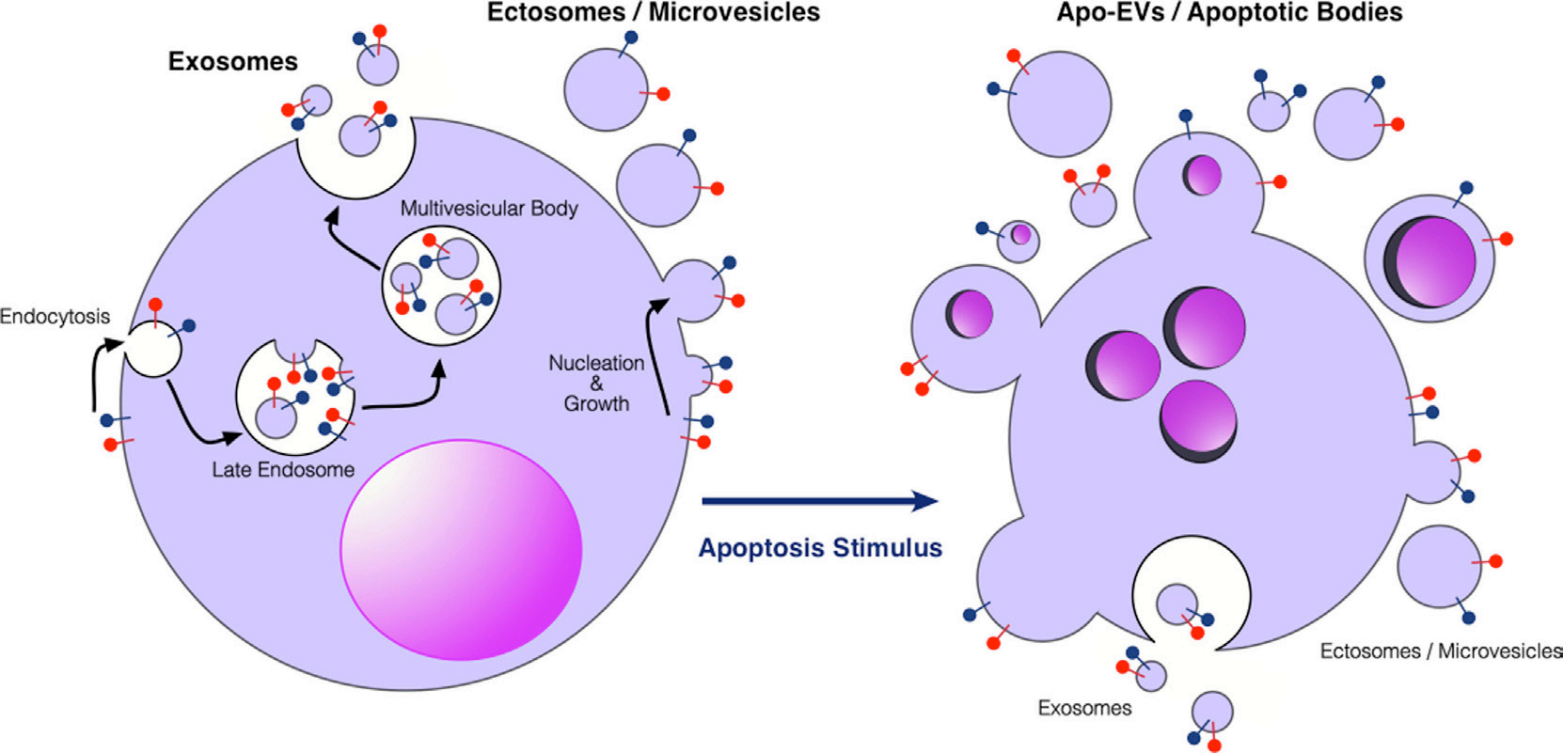
Extracellular vesicles (EVs) produced by viable and apoptotic cells. Diagrammatic representation of the broad categories of EVs produced by viable cells (left) and cells undergoing apoptosis (right). Exosomes are produced through endosomal pathways involving the formation of multivesicular bodies that fuse with the plasma membrane. Ectosomes or microvesicles bud directly from the plasma membrane. Apo-EVs are produced in a diverse size range specifically by apoptotic cells—larger Apo-EVs are commonly known as apoptotic bodies. Apo-EVs can contain large cellular organelles including nuclear fragments as shown. Relatively little is yet known about the molecular mechanisms underlying the production of Apo-EVs/apoptotic bodies and the roots of their heterogeneity [see Ref. (18, 27), for reviews] or indeed their relationships with exosome or ectosome production. Preparations of Apo-EVs are likely to be contaminated by exosomes and ectosomes produced at least during pre-apoptosis stages of activation. Red and blue “lollipop” symbols serve to illustrate orientation of transmembrane proteins of the plasma membrane. Left-hand cartoon adapted from Ref. (17).

EVs are subcellular membrane-delimited particles, which can be released from cells both constitutively and in response to activation or stress. Although there is a lack of consensus on properties and nomenclature within the EV field, it is generally accepted that there are at least three different types of EVs, which have been classified according to size, biogenesis, or isolation technique (12–18) (Figure 1). Exosomes are the smallest category (30–150 nm) formed from endosomal membranes and released from the cell by exocytosis of multivesicular bodies. Ectosomes or microvesicles (100–1,000 nm) bud directly from the plasma membrane. Apoptotic bodies (typically described as 1,000–5,000 nm) are formed during apoptosis, and apoptotic cell-derived vesicles have very broad size ranges (our unpublished observations). Relatively large EVs (ectosome-like), >2 μm in diameter and not apparently associated with apoptosis, have also been described, showing intact organelles but not nuclear components [see, for example, Ref. (19)]. EV cargoes are diverse, ranging from nucleic acids such as miRNA, mRNA, and DNA, to lipids, and cytosolic and membrane proteins. It is thought that the sequestering of specific cargoes into EVs is directed, but the mechanisms by which this occurs have not yet been fully elucidated. EVs can be detected in most bodily fluids, including blood, urine, saliva, amniotic fluid, breast milk, and ascites. There has been a growing interest in using circulating EVs as carriers of biomarkers of disease, especially since EVs carry markers of their cell of origin and may represent the pathophysiological status of the cells (12–17, 20–22). EVs are involved in the regulation of tumor growth, progression, and antitumor immunity, in the latter context showing potential application in antitumor vaccination [see Ref. (23–26) and forthcoming Frontiers in Immunology Research Topic: *The Immunomodulatory Properties of Extracellular Vesicles from Pathogens, Immune Cells and Non-Immune Cells*].

EVs both from tumor cells and from stromal cell components of the tumor microenvironment can promote cancer growth and metastasis. Accruing evidence indicates that the pro-oncogenic properties of EVs are mediated *via* multiple mechanisms, reflecting the multitude of cargoes and the complex biological composition of EVs. For example, tumor cell-derived EVs can promote tumor growth and angiogenesis through transferral of mutant receptors, angiogenic proteins, and RNAs from tumor cells to neighboring cells, including endothelial cells and mutant growth receptor-deficient tumor cells in the tumor microenvironment (22, 28, 29). EVs can promote tumor metastasis through horizontal transfer of oncogenic molecules from cancer cells to bone marrow-derived stromal cells (30) or directly from malignant tumor cells to relatively benign counterparts, endowing the recipient cells with metastatic properties (31). EVs from tumor cells also endow stromal cells with switched metabolic pathways (32) and can alter the activation status of fibroblasts to resemble that of cancer-associated fibroblasts (CAFs) (33, 34). Furthermore, EVs derived from the stromal or immune cells of tumors have regulatory properties in cancer. For example, M2-polarized tumor-associated macrophages (TAMs) have been shown to transfer microRNA-21 to gastric cancer cells, suppressing apoptosis, and thereby causing cisplatin resistance (35).

## Apoptotic Tumor Cell-Derived EVs

Apoptotic cells in tumors communicate with neighboring cells not only by intercellular contact but also *via* soluble and EV-encapsulated signal mediators (36, 37). EVs from apoptotic cells display a broad size heterogeneity from around 50 nm to several microns and the term “apoptotic body” is often used to describe the larger varieties—commonly >1,000 nm—of apoptotic cell-derived EVs (Apo-EVs) (18). However, the terminology describing the different types of membrane-delimited subcellular-sized particles released from apoptotic cells is currently a matter of discussion, as a standardized nomenclature has not been established to date (38). We favor the concept that Apo-EVs represent a continuum (albeit heterogeneous) of vesicles released from apoptotic cells with wide variation in size, including those classed as apoptotic bodies (Figure 1). Although there seems little doubt that Apo-EVs will prove to be heterogeneous in other ways [e.g., some carrying genomic DNA and/or organelles such as mitochondria, together with heterogeneity in macromolecule content (39–42)], the critical definition of an Apo-EV is a vesicle that is apoptosis dependent. Clearly EVs may be released from apoptotic cells as a consequence of pre-apoptosis stress signals or as a result of post-apoptotic necrosis. The need for more information about the EVs released throughout the apoptotic process is reinforced by evidence of significant levels of proteins such as histones that are loaded into Apo-EVs prior to the loss of plasma membrane integrity (40). Since Apo-EVs encapsulate a wide variety of bioactive molecules and cellular organelles (39–43), they can be characterized as metabolically active structures that provide apoptotic cells with the ability to transduce signals over relatively long distances (6, 36, 37, 44).

Although several studies have been forthcoming in recent years, the structural characteristics, contents, and functional attributes of Apo-EVs in cancer remain poorly defined (6, 18, 44), particularly since apoptosis dependence of putative Apo-EVs has not been stringently investigated, for example, by comparing EVs from apoptotic and non-apoptotic cells subjected to identical stress signals. Notwithstanding this limitation, we highlight the potential for Apo-EVs and their cargoes to play important roles in the regulation of tumor growth and progression. Thus, it has been shown that cancer cells under stress transfer genetically active material to their neighbors and given that EVs are rich in nucleic acids—including both DNA and RNA [the former likely to be especially enriched specifically in Apo-EVs (our unpublished observations)]—they are likely to play a significant role in this communication (45). Furthermore, stromal cell-derived EVs (<100 nm) released as a consequence of cell stress may provide key signals supporting the neighboring tumor cells’ capacity to metastasize, promoting proliferation and inhibiting apoptosis (46). Immunological functional heterogeneity of Apo-EVs is evident from studies indicating that, on the one hand they can be immunosuppressive (26), while on the other (albeit in a different context), immunostimulatory (41). Horizontal transfer of potentially oncogenic genomic DNA through phagocytosis of apoptotic bodies and its control through p53-mediated DNA damage responses has been demonstrated (47–49), raising the possibility that cells receiving Apo-EVs may, even transiently, express gene signatures from apoptotic tumor cells, with implications for tumor growth. It is also conceivable that oncogene transfer through Apo-EVs could lead to sustained transformation of recipient cells. Indeed, the production of EVs by tumor cells in response to anticancer therapies—which are well known to induce apoptosis in tumor cells—suggests that EV production is detrimental to therapeutic success (50–52). Mechanisms of EV-mediated drug resistance are beginning to emerge. For example, in pancreatic ductal adenocarcinoma (PDAC), EVs produced by CAFs as a consequence of chemotherapy transfer Snail and miR-146a to PDAC cells, enhancing both their survival and proliferation during treatment (53). Further work will clarify roles specifically for Apo-EV in tumor growth and progression pre- and posttherapy.

## The Onco-Regenerative Niche (ORN)

Our group has proposed that Apo-EVs are key components in a conceptual tissue repair microenvironmental “module” we have termed the *ORN* (6, 44). This concept is based upon the well-rehearsed comparison of tumors to “wounds that fail to heal” or, perhaps more accurately, “wounds that fail to stop repairing.” We propose that the ORN represents a microenvironmental signaling network driven by apoptosis and involving tumor cells, non-tumor stromal and immune cells, connective tissue, soluble factors, and EVs. The putative network engenders pro-repair and regenerative responses that promote tumor cell proliferation, angiogenesis, and invasiveness while at the same time suppressing antitumor immunity. Currently, the ORN remains conceptual, serving as a platform for rationalized experimentation to test the hypothesis that conserved tissue repair and regenerative responses to cell death in tumors provide fundamental pro-oncogenic signals that can facilitate tumor growth, metastasis, and posttherapeutic relapse.

**Figure 2:**
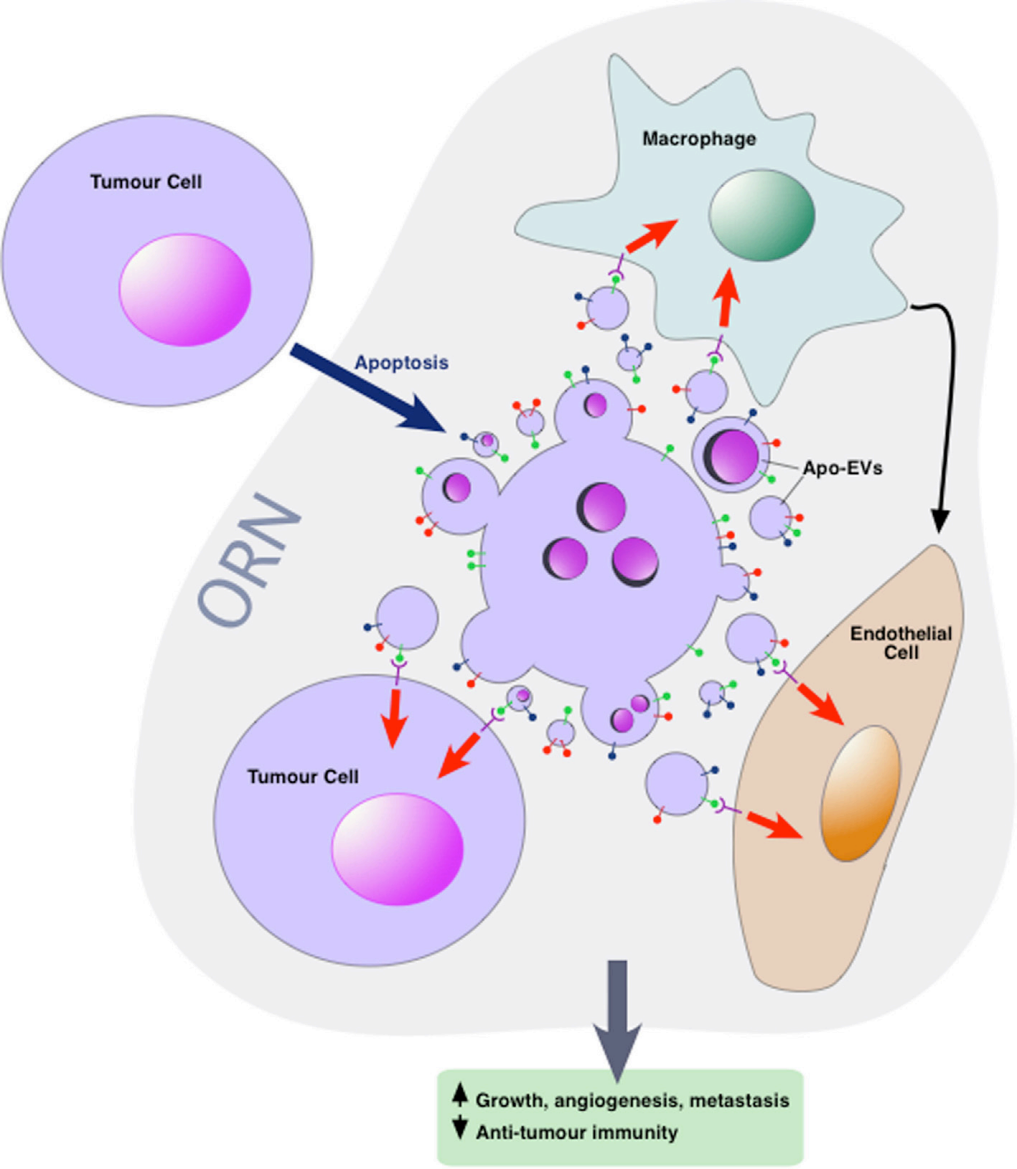
Apoptotic tumor cell-derived extracellular vesicles (Apo-EVs) in the onco-regenerative niche (ORN). Cartoon depicting the potential involvement of Apo-EVs in providing key intercellular networking and activating elements of the ORN. Currently, the ORN is conceptualized as an apoptosis-driven oncogenic niche. Here, this is represented by Apo-EVs communicating with non-tumor cellular elements of the niche such as tumor-associated macrophages and endothelial cells as well as with viable tumor cells. Although little is yet known of the modes of interaction of Apo-EVs with recipient cells, it seems reasonable to assume that at least some of the molecules [see text and Ref. (57)] involved in clearance of apoptotic cells (and relatively large apoptotic bodies) will prove to be shared by smaller varieties of Apo-EVs. In this context, it is noteworthy that receptor-mediated cellular interaction of EVs involves the phosphatidylserine receptor TIM-4 and the integrins αvβ3 and αvβ5 (58), all of which are well defined as phagocyte receptors for apoptotic cells. Novel pathways also seem likely. Here, the surface of Apo-EVs is shown to display signals (green “lollipop” symbols), putatively involved in engagement with appropriate receptors on cells of the niche. We propose that, in addition to being targeted for degradation in phagolysosomes, Apo-EVs may transfer cargoes, including surface receptors and nucleic acids, to recipient cells, as has been described for other EV types, thereby altering cellular functional activities (red arrows) and intercellular communication (e.g., black arrow), culminating in the pro-oncogenic activities of the ORN such as the promotion of clonal tumor cell growth, angiogenesis, and metastasis, together with inhibition of antitumor immunity.

Intercellular communication events concerning apoptotic cells, which lie at the heart of the ORN, involve TAMs, endothelial cells, and viable tumor cells among others including additional immune cells, CAFs, and mesenchymal stromal cells. Little is yet known about the mechanisms by which Apo-EVs interact with neighboring cells of the tumor microenvironment although EVs released by apoptotic cells harbor “find-me” signals that facilitate the directed migration of phagocytes to apoptotic cells. In some studies, active chemotactic molecules associated with EVs (CX_3_CL1 and ICAM-3) have been described (54–56). In the context of phagocytosis, apoptotic cells are able to activate multiple lineages of cells in addition to macrophages, including dendritic cells, epithelial cells, bone marrow stromal cells, muscle cells, fibroblasts, and endothelial cells. Although direct cell-to-cell contact is an obviously essential prerequisite for phagocytosis of apoptotic cell bodies, information is currently limited on the potential roles of Apo-EVs in activating the phagocytes of apoptotic cells. Given the multiple modes by which EVs can interact with cellular targets, we speculate that both receptor-dependent and independent mechanisms operate in the ORN to provide communication pathways between apoptotic cell-derived EVs and other cells in the microenvironment—both phagocytes and non-phagocytes, non-transformed and transformed cells (Figure 2). First-line candidate receptors would be those already known to function in apoptotic cell clearance, especially the phosphatidylserine (PS)-binding glycoproteins, be they opsonins such as Gas6, Protein S, or MFG-E8, or cell surface PS receptors such as TIM-4, BAI-1, and Stabilin 2 [reviewed recently by Ref. (57)], especially since the majority of EVs, including those released from apoptotic cells tend to expose PS [(12) and our unpublished observations]. Additional, PS-independent mechanisms may also be involved, and mechanisms of activation of the cellular targets of Apo-EVs would be expected to include agonistic or antagonistic receptor ligation, localized release or transfer of highly labile, biologically active molecules and transfer of proteins, nucleic acids, and metabolites as has been described for EVs in other contexts.

Although limited mechanistic information is yet available, we propose that EVs released into the tumor microenvironment have multiple tumor-modulating activities. Apoptotic cells activate diverse responses in neighboring cells, producing mitogens, anti-inflammatory mediators, pro-angiogenic factors, and matrix-degrading enzymes in addition to chemoattractants (6, 10, 44). Given their biological potential as we have already discussed, it seems likely that Apo-EVs will prove to harbor key bioactive molecules that mediate at least some of the aforementioned tumor-modulating activities.

## Clinical Potential of Apoptotic Tumor Cell-Derived EVs

It is widely accepted that EVs contain biomolecules indicative of the cell from which they derive, its state of activation, its metabolic activity, and in some cases its genotype. Since EVs are accessible in various body fluids, their cargoes may be useful as non-invasive biomarkers for diagnosis and prognosis of disease, including cancer. Recent investigations serve to highlight the association of EVs and their cargoes with diverse cancer types. For example, a recent study of EVs isolated from blood of colorectal cancer patients, based on EpCAM^+^ selection, found increased levels of specific miRNAs in patients compared to healthy controls, which decreased after surgical removal of the tumor (59). EVs from patients with liver cancer (hepatocellular carcinoma and cholangiocarcinoma) are measurable and distinct from EVs derived from non-cancerous chronic liver disease such as cirrhosis (60). A study of multiple myeloma showed that the number of EVs expressing the plasma cell marker CD138 in the blood of patients corresponded to both disease state and therapeutic outcome, giving both diagnostic and prognostic relevance to the detection of EVs in this disease (61). In PDAC patients, mutant *KRAS* DNA can be detected with a higher frequency in circulating EVs than the more prevalent method of cell-free circulating DNA (62). Detection of miRNA in the EVs of Hodgkin lymphoma patients was followed along the course of treatment and was found to decrease concordantly with the FDG-PET scans that are routinely used to monitor this cancer type, and in patients with relapsing disease those miRNA levels were seen to rise (63). In breast cancer, the TRPC5 receptor channel, shown to be essential for chemoresistance, can be detected on circulating EVs and used to predict treatment response and relapse (64). In prostate cancer, two proteins—ADSV and TGM4—found in urinary EVs, can in combination predict not only the presence of the disease but also relapse of the patient after treatment (65).

These examples illustrate that there is conceptual concordance between the biological activities of EVs in mechanistic cancer models and their association with disease status in human patients. As yet, however, the particular significance of Apo-EVs in human malignant disease remains unclear. We speculate that a fraction of the Apo-EVs from cells of the tumor microenvironment, including tumor cells themselves, macrophages, and other immune cells, together with fibroblasts, mesenchymal stem cells, and endothelial cells, would gain access to the circulation [note the well-established circulation of tumor cells and nucleosomes in cancer patients (66, 67)] and as such would be readily amenable to analysis using appropriate technologies (*vide infra*) and could potentially provide useful diagnostic and prognostic information of benefit to patient care. As well as providing valuable cargo information, EVs (and possibly Apo-EVs too) may also antagonize cancer therapies by acting as “decoy” vehicles for therapeutic antibodies, thereby diluting effective biopharmaceutical delivery to tumor cells. This has been shown to be the case for the anti-CD20 therapeutic antibody Rituximab, which binds EVs produced by lymphoma cells, effectively protecting the cells from immunotherapeutic destruction (68). Similarly, EVs from HER2^+^ breast cancer carry HER2 molecules that can bind to the anti-HER2 therapeutic antibody, trastuzumab, compromising its effectiveness (69). Thus, EVs, possibly including Apo-EVs, are capable of inactivating therapy as well as promoting tumor growth, and so reducing EV production may provide a novel strategy to improve therapeutic responses.

## Conclusion

Although EVs, including Apo-EVs, show much promise for use both in diagnosis and treatment monitoring, and potentially even as therapeutic targets, there are many hurdles to overcome before this can be translated effectively to the clinic. Perhaps the most significant challenge is EV heterogeneity and lack of standardization in terms of EV isolation and characterization in clinical samples (or indeed any samples). Much research effort is focused on purification of EV populations using advanced platforms such as immunomagnetic isolation and immunoaffinity arrays (70, 71), which also hold potential to become miniaturized, enabling the development and application of point-of-care technologies in the clinical setting. Methods of characterization are also a challenge, as the size of the smallest EVs pushes many techniques beyond their lower limits of reliability. However, progress is likely to be speedy, following the rapid progress in nanometrology that has occurred in recent years. The relatively recent explosion in EV interest and research has led to significant progress, not least in clinical oncology diagnostics (see, for example, products from Exosome Diagnostics http://www.exosomedx.com).

Because of their structure and highly specific cargoes, EVs are likely to prove to be most useful targets for biomarker screening in cancer, as well as in other disease settings. It is anticipated that new technologies will allow screening of tiny, readily accessible biopsy samples, most commonly blood, and that the vesicular nature of EVs will provide for concentration and protection of valuable biomarker cargoes, including labile molecules. Resolution of the phenotypic and functional heterogeneity of EVs is already under way, and we predict that substantial advances will continue to be made in EV research in the near future, not least in the functional attributes of Apo-EVs and their relevance to human cancer diagnosis, prognosis, and therapy.

## Author Contributions

All authors planned and cowrote the manuscript.

## Conflict of Interest Statement

The authors declare that the research was conducted in the absence of any commercial or financial relationships that could be construed as a potential conflict of interest.
